# Objective Assessment of Adherence and Inhaler Technique among Asthma and COPD Patients in London: A Study in Community Pharmacies Using an Electronic Monitoring Device

**DOI:** 10.3390/pharmacy11030094

**Published:** 2023-06-04

**Authors:** Iman Hesso, Shereen Nabhani-Gebara, Reem Kayyali

**Affiliations:** School of Life Sciences, Pharmacy and Chemistry, Kingston University London, Penrhyn Road, Kingston upon Thames, London KT1 2EE, UK; i.hesso@kingston.ac.uk (I.H.); s.nabhani@kingston.ac.uk (S.N.-G.)

**Keywords:** electronic monitoring device, adherence, inhaler technique, community pharmacists, acceptability, asthma patients, COPD patients

## Abstract

Background: The INhaler Compliance Assessment (INCA^TM^) device is an electronic monitoring device (EMD) that assesses both patient’s adherence and inhaler technique (IT). This study aimed, first, to assess the value of using the INCA^TM^ device as an objective measure during medicine use review (MUR) consultations provided by community pharmacists (CPs) on patients’ adherence and IT. Second, we aimed to explore patients’ perceptions about the INCA^TM^ device. Methods: A mixed methods approach was used, involving two phases. Phase one was a service evaluation in independent community pharmacies in London with a before-and-after study design. The service included provision of an MUR consultation to asthma and COPD patients using objective feedback about adherence and IT generated with the INCA^TM^ device. Descriptive and inferential statistics were performed using SPSS. Phase two involved semi-structured interviews with respiratory patients. Thematic analysis was performed to generate key findings. Main findings: Eighteen patients participated in the study (12 COPD and 6 asthma). The results showed significant improvement in the INCA^TM^ actual adherence from 30% to 68% (*p* = 0.001) and significant reduction in IT error rate from 51% to 12% (*p* = 0.002) after conducting the service. Analysis of the interviews revealed patients’ positive attitudes in terms of the perceived benefits of the technology and a desire for future use and recommendation for others. Patients had also positive attitudes towards the consultations provided. Conclusion: Embedding an objective measure about adherence and IT during CPs’ consultations showed a significant improvement in patients’ adherence and IT and was accepted by patients as well.

## 1. Introduction

Assistive digital technologies that are designed to promote all types of chronic disease management are expected to expand in the future [1]. In the field of respiratory medicine, problems pertaining to poor inhaler adherence and poor inhaler technique (IT) have been extensively reported in the literature among respiratory patients [2,3], with no evidence of improvement in both aspects to date [2,4,5]. Assistive technologies such as electronic monitoring devices (EMDs) have been increasingly employed to assess adherence to inhaler therapy in a more objective and accurate way, compared to the other traditional methods such as self-reporting, canister weighing, dose-counting, and pharmacy records, which are fraught with limitations in terms of subjectivity and inaccuracy [2,6,7,8,9,10,11,12,13]. Several randomised controlled trials (RCTs) [14,15,16,17,18,19,20,21] and clinical studies [22,23] were conducted throughout the last decade and demonstrated significant improvements in inhaler adherence with the use of different EMDs. The INhaler Compliance Assessment (INCA^TM^) device is amongst the few emerging EMDs that objectively measure both adherence and IT at the same time while patients are using inhalers at home. It is an acoustic, battery-operated device that can be mounted on the top of the dry powder inhaler (DPI) without interference with how the drug is delivered from the inhaler or the way the inhaler is used [2,11,24]. Details about the design of the device and its validation have been previously published [2,10,11,24,25,26,27,28,29]. The device records the audio associated with inhaler usage and the date and time of inhaler use as well. Analysis of the audio files is performed through an automated algorithm, thus providing graphical and written feedback about the patient’s IT and adherence [30]. The device has been certified by the European Union (EU registered) and approved by the Food and Drug Administration (FDA) [31,32]. The device is manufactured by Vitalograph Ltd. [11,24,25,28].

However, the success of health technologies depends mainly on their acceptability and perceived usefulness by end users [1,6,33]. In fact, there has been a concerted call for the need to promote users’ involvement, including patients and healthcare professionals (HCPs), in the design and implementation of new technologies in healthcare to ensure their success [1,6,34]. Nevertheless, and despite increased use of EMDs for inhaler therapy in adherence research and clinical practice nowadays [34], there is a scarcity of research into patients’ perceptions of these devices [6]. Furthermore, most research published to date [1,6,33] has been conducted to elicit patients’ perceptions about EMDs that assess inhaler adherence only.

Therefore, the main aims of this research were first to assess the value of using the INCA^TM^ device as an objective measure for adherence and IT during a generic medicines use review (MUR) delivered by community pharmacists (CPs) on patients’ adherence and IT. Second, we aimed to explore patients’ perceptions about the use and acceptability of the INCA^TM^ device, as an example EMD for the assessment of both adherence and IT.

## 2. Material and Methods

A mixed research method involving two phases was used. Phase one was a service evaluation of an MUR consultation using the feedback generated by the INCA^TM^ device, whereas phase two was a qualitative study to explore patients’ perceptions regarding the used EMD.

### 2.1. Phase One: Service Evaluation

This phase was undertaken to address the first aim. This was a prospective, multi-site, service evaluation in community pharmacies within West and South London, using a before-and-after design with the subjects serving as their own control. The service evaluation was preceded by an observational study, which examined the level of adherence and IT among 48 asthma and COPD patients using the INCA^TM^ technology [2], hence providing baseline data for participants in this study. Details about the observational study have been previously published [2]. Thirteen out of the 23 CPs who participated and recruited patients in the observational study were able to recruit patients for the service evaluation. All patients recruited for this study have already participated in the observational study, with no new patients being recruited. Patients were approached irrespective of their feedback results in the observational study. The current study was conducted between June 2017 and January 2018. The purpose of this study was explained to the patients by the CP. Patients were informed that they will have a discussion with their CP about the IT and adherence of their maintenance inhaler (salmeterol/fluticasone DPI) while using the feedback generated from the INCA^TM^ device. After the discussion with the CP took place, patients were informed that they had to fill a feedback questionnaire about the provided consultation and that an INCA^TM^ device will be attached to their DPI by one of the research team (IH) to obtain further feedback about adherence and IT after the consultation. Patients were asked to return their inhaler with the mounted INCA^TM^ device (the adapted accuhaler) once finished along with the feedback questionnaire in a sealed envelope. Patients who agreed to participate were included. The researchers were constrained to a small sample size for several reasons, including available time, logistical reasons, and limited resources.

#### 2.1.1. Outcome Measures and Calculations

**INCA^TM^ objective adherence:** adherence to salmeterol/fluticasone accuhaler was assessed before and after the service evaluation using the INCA^TM^ device to provide quantitative and objective information about the patient’s inhaler usage during the one-month period after the discussion with the CP. INCA^TM^ adherence rates (actual and attempted) were calculated as an area under the curve (AUC). Details about adherence calculation were previously described and published [35]. Attempted doses refers to doses that patients attempt to take, i.e., opened the inhaler, primed the device, and attempted to inhale, whether doses are taken correctly or incorrectly. Conversely, actual doses refer to doses taken by patients with no technique errors [35]. Based on the literature [36,37,38,39], a cut-off point of ≥80% indicates good adherence.

**Inhaler technique:** IT was measured in a quantitative way before and after using the INCA^TM^ device and is calculated and reported as a technique error rate (TER). Details about TER calculation were detailed previously [2], with a TER of ≥20% indicating a form of poor IT [2].

**Patient satisfaction:** this was assessed using a questionnaire (Supplementary File S1) designed by the authors to elicit the participants’ feedback about the device ergonomics, in terms of usage and handling, and different aspects of the consultation provided, including information and feedback provided during the consultation, and consultation style. The questionnaire consisted of 13 questions: 12 were rating questions on a 5-point Likert scale and the last question was a free-text question for any further comments. A pilot study was conducted with 5 patients who only participated in the observational study for face and content validation of the questionnaire. For face validity, participants were asked about the questions’ clarity and ease of comprehension. Content validity focused on completion of the questionnaire to ensure that the designed tool adequately contained all the relevant questions to measure patient satisfaction to ensure that appropriate results could be deduced from the designed tool. No further changes were required for the questionnaire as suggested by the results of the pilot study. The results of this pilot study were not included in the final analysis.

#### 2.1.2. Data Analysis

Descriptive statistics were conducted to describe patient’s characteristics, errors in inhaler use, and questionnaire results. Due to the small sample size, continuous variables were expressed using median score and inter quartile range (IQR). Proportions/percentages were used to describe variables that were categorical in nature. In addition, frequencies were used to describe the distribution of different IT errors. Non-parametric tests were performed due to the small sample size of the study; thus, comparisons of repeated measures of continuous variables were performed using the Wilcoxon signed-rank test. Where applicable, the comparison of proportions of participants with repeated measures was performed using McNemar test. SPSS version 24 was used to conduct the analysis, with statistical significance being set at *p* < 0.05 for all analyses.

### 2.2. Phase Two: Semi-Structured Interviews Regarding Perceptions and Acceptability of the INCA^TM^ Device among Patients (the Qualitative Phase)

This phase was conducted to address the second aim. Patients who had the service evaluation were also invited by the CP to have a brief interview with one of the research team after the discussion. Patients who accepted to participate were included.

#### 2.2.1. Data Collection

The interviews were conducted in person by the first author in the private consultation room at the pharmacy to avoid distraction and maintain confidentiality. Eighteen patients were interviewed between June 2017 and January 2018. All interviews were audio-recorded and handwritten notes were taken during the interviews. The interviews lasted between 5 and 24 min.

#### 2.2.2. Interview Topic Guide

The interview schedule was developed by the authors (Supplementary File S2) and consisted of 9 open-ended questions, covering three main sections. The first section was developed to elicit the opinions of patients about the consultations provided by the CP. The second section aimed at eliciting information about the use of the INCA^TM^ device and the feedback generated in terms of understanding, format, and content. The patients were shown their feedback again during the interview to guide and facilitate the interview. The third section aimed to explore patients’ suggestions for improving the consultation provided. At the end of the interview, patients were asked to discuss any additional information/comments that they feel were important but not covered during the interview.

#### 2.2.3. Data Analysis

Interview notes and recordings were transcribed verbatim and analysed by the first author. Thematic analysis using inductive/deductive approaches was employed to generate key themes and subthemes [40]. Data familiarisation was achieved through iterative reading of the interview transcripts, which allowed the identification of potential emergent codes. The coding of the transcripts was facilitated using NVivo 11 software. The coding and interpretation of themes and associated subthemes was a recursive process which involved extensive review and modification of the emergent themes and subthemes [40]. The identified codes were continually checked, discussed, and confirmed with the research team to enhance analytical rigour and avoid bias. Results were presented in the form of themes and subthemes where relevant. Direct quotations were included within each theme and subtheme to substantiate the findings.

### 2.3. Ethical Consideration

The Research Ethics Committee at Kingston University considered the study as service evaluation. The authors also checked the NHS Health Research Authority (HRA) decision tool, which indicated no need for ethical approval. Ethical approval for interviews was obtained from the Research Ethics Committee at Kingston University London (Ref. No.: 1415/043).

## 3. Results

### 3.1. Service Evaluation

#### 3.1.1. Study Population and Demographics

Eighteen out of the 48 patients who were recruited for the observational study participated in the service evaluation. The median age of participants was 64.5 years; 67% had COPD and 33% had asthma. Patients were prescribed a median of 10 medications. The majority of patients did not have accident and emergency (A&E) visits or hospital admissions due to their respiratory condition in the previous year. However, more than half (56%) had exacerbations in the preceding year. Patients’ demographics are presented in Table 1.

#### 3.1.2. Level of Adherence among Patients before and after the MUR Type Consultation with the CP

All the 18 patients had audio recordings at baseline and after the consultation. The number of doses expected to be taken, in an ideal situation for one month, is 1080 doses (18 × 60 = 1080 doses). At baseline, the total number of doses taken according to the dose counter was 1072; however, there were 999 audio files of attempted doses recorded by the EMD (*p* = 0.756). After the consultation with the CP, there were 1077 doses taken as indicated by the dose counter and 1077 audio files of attempted use by the EMD. Patients were instructed to use and return their adapted accuhaler after 1 month. However, due to poor adherence, many patients continued to use their inhaler after the ideal 30 day period. Therefore, audio files recorded after the 1-month period were excluded from the analysis. Adjusting for 30 days usage, there were 830 audio recordings with attempted use at baseline compared to 907 after the consultation with the CP (*p* = 0.421) (Table 2).

Of the 60 doses expected to be taken monthly per patient, a median of 50 (83.34%) doses were attempted at baseline compared to 51 (85%) doses after the CP’s consultation (*p* = 0.421). When all IT errors were included in the calculations (this involved subtracting doses with IT errors from the total doses), the median number of actual doses (i.e., doses with no IT errors) was 22.5 at baseline and 44 after the CP’s consultation (*p* = 0.002), indicating a statistically significant increase in the number of actual doses after the consultation.

The median INCA^TM^ attempted adherence for the 18 patients at baseline was 68% (IQR: 30). Interestingly, after the consultation with the CP, the median INCA^TM^ attempted adherence became 80.5% (IQR:16), indicating a statistically significant change in attempted adherence between baseline and after the consultation (*p* = 0.019). At baseline, only 22% of patients (*n* = 4/18) had an attempted adherence ≥ 80%, whereas, after the consultation, 56% of patients (*n* = 10/18) had an attempted adherence ≥ 80% (Table 3). The median INCA^TM^ actual adherence at baseline for the 18 participants was 30% (IQR: 37.5), whereas the median INCA^TM^ actual adherence after the consultation became 68% (IQR: 23.75), indicating a statistically significant improvement in actual adherence between baseline and after the consultation (*p* = 0.001). The objective data provided by the INCA^TM^ device showed that none of the 18 patients had an actual adherence rate ≥ 80% at baseline. However, after the discussion with the CP, 22% of patients (*n* = 4/18) had an actual adherence rate ≥ 80% (Table 3).

#### 3.1.3. Level of Inhaler Technique among Patients before and after the Discussion (Service Evaluation) as Determined by the INCA^TM^ Device

The median TER per patient at baseline was 51% (IQR: 72). Interestingly, the median TER became 12% (IQR: 29) after the consultation, indicating a statistically significant reduction in TER between baseline and after the consultation (*p* = 0.002).

Analysis of audio files at baseline indicated that 396 (48%, *n* = 396/830) files exhibited correct IT and 434 (52%, *n* = 434/830) had errors in IT in the one-month period. However, after the CP’s consultation, analysis of audio data showed that 726 (80%, *n* = 726/907) files had the correct technique of inhaler use (*p* = 0.002, compared to baseline data) and only 181 (20%, *n* = 181/907) had errors in the technique during the month (*p* = 0.003, compared to baseline data). The median number of errors per patient in a one-month period was 19.5 at baseline (IQR: 31.25), but this was significantly reduced to 6 (IQR: 13.25) after the CP’s consultation (*p* = 0.003).

At baseline, errors in drug priming (i.e., either multiple priming, failure to prime the device correctly, or dose dumping) accounted for 19.4% (*n* = 84/434) of all errors. Errors in inhalation represented 80.6% (*n* = 350/434) of all errors (Table 4). Multiple inhalations was the most dominant error, occurring in 216 (49.7%) of all events, followed by drug priming without subsequent inhalation, which represented 28.6% (*n* = 124/434) of all errors, and lastly failure to prime the inhaler correctly, which was seen in 75 (17.3%) events (Table 4). After the consultation with the CP, errors in drug priming accounted for 28.2% of all errors, while errors in inhalation represented 71.8% of all errors (Table 4). Multiple inhalations was still the most frequent error, occurring in 46.4% (*n* = 84/181) of all events. However, the second most prevalent error was failure in priming the device correctly, which was recorded in 45 (24.9%, *n* = 45/181) events, and lastly drug priming without subsequent inhalation, which accounted for 23.8% (*n* = 43/181) of all errors (Table 4). There was a reduction in the number of errors across all types after the consultation compared to baseline. However, the reduction was only significant for the error related to multiple inhalations (Table 4).

There was a significant difference in the proportion of patients with a TER ≥ 20% at baseline and after the consultation (*p* = 0.039). More than three quarters of the patients (78%) at baseline had a high TER (TER ≥ 20%) within a one-month period compared to 33% after the consultation (Table 5).

#### 3.1.4. Feedback Questionnaire Data

Feedback about the use of the INCA^TM^ device (device ergonomics)

More than two-thirds of patients (67%, *n* = 12/18) found the accuhaler with the mounted INCA^TM^ device very easy to use, 5 patients found it easy (28%), whereas only one patient (5%) found it neither easy nor difficult. Hence, none of the patients found the use of the adapted accuhaler difficult or very difficult. As for the handling of the adapted accuhaler, 61% (*n* = 11/18) of patients responded favourably, rating it as very manageable, and 39% (*n* = 7/18) as “manageable. None rated it as cumbersome or very cumbersome.

Feedback about the consultation

With respect to the counselling information, all patients provided positive rating, with 72% (*n* = 13/18) rating it as very easy and 28% (*n* = 5/18) rating it as easy. Similar results were obtained regarding the INCA^TM^ feedback, since 61% (*n* = 11/18) rated the feedback as very easy and 39% (*n* = 7/18) rated the feedback to be easy. The consultation style of the CP was rated as very easy (72%, *n* = 13/18) or easy (28%, *n* = 5/18) by the patients. The language used during the consultation was found very easy by half of the patients (50%, *n* = 9/18), and easy by 8 patients (44%, *n* = 8/18). Only one patient rated this as neither easy nor difficult.

Regarding the usefulness of the counselling information, most patients (*n* = 17/18) had positive responses, either strongly agree (61%, *n* = 11/18) or agree (33%, *n* = 6/18). Only one patient was undecided about this aspect. As for the usefulness of the INCA^TM^ feedback, 61% (*n* = 11/18) strongly agreed and 39% (*n* = 7/18) agreed to its usefulness. Thus, all patients perceived the INCA^TM^ feedback to be useful.

### 3.2. Semi-Structured Interviews

Semi-structured interviews were conducted face-to-face with all the 18 patients. Thematic analysis generated 4 main themes with relevant subthemes (Table 6).

#### 3.2.1. Acceptability of the INCA^TM^ Technology

Patients’ positive perceptions about the INCA^TM^ technology

All interviewees were quite receptive to the idea of monitoring their performance and were willing to use the INCA^TM^ device again and get further feedback about their adherence and IT in order to see the difference and to ensure that they are using their inhaler correctly after the consultation with the CP.

*“Yes, because although I know how to change the technique now, I want to be 100% sure I am getting it right next time”*.

(Patient 6)

Interestingly, one participant suggested the incorporation of a mechanism which can show the patient instantly if the inhaler is used correctly or not, as highlighted in the following excerpt.

*“If there is a way that the device tells you whether you are doing it right or wrong when you are taking it, like a green colour if used correctly or red colour when used wrongly”*.

(Patient 9)

Recommendation of the INCA^TM^ device to other patients

All patients advocated the recommendation of the INCA^TM^ device to other patients using inhalers. This is because the use of the INCA^TM^ device would be helpful and beneficial to other patients, who might be in a similar situation, in identifying how they are really using their inhalers, especially given that many of the interviewees were thinking that they have good performance, which was not the case as identified by the INCA^TM^ device.

*“Well I would suggest it to them (referring to other patients) because I was thinking I was doing everything correctly when I wasn’t so may be it would be helpful for them to learn if they are doing it correctly or not”*.

(Patient 18)

Some patients suggested rolling-out the INCA^TM^ technology to other patients with respiratory conditions, after seeing their results, in order to make other patients aware of their performance.

*“I just hope that this could be rolled out to many more asthma patients…I can be a live testament”*.

(Patient 12)

Interestingly, some patients supported the use of the INCA^TM^ device to other patients from a different angle by indicating that monitoring inhaler usage for a short period of time for reassurance will not be harmful, especially given that the INCA^TM^ device does not affect the inhaler usage.

*“Absolutely, absolutely yes, because I am surprised that I am not using the inhaler properly…. and if I come at that the reverse angle is to say what harm can you do for somebody to check for a short period if their technique is okay”*

(Patient 16)

#### 3.2.2. Patients’ Misperceptions about Their Inhaler Usage

What was obvious from the interviews is that patients were overestimating their own performance; some patients articulated their astonishment upon seeing the feedback because they thought they had been using their inhaler correctly for years.

*“I thought I was using that inhaler properly (the patient laughs calmly) but I was not obviously… I am just shocked that I haven’t used it properly not even once…. I mean how many years I have been taking this now. 10 years”.*

(Patient 7)

*“…this one (referring to graph related to IT) is actually more shocking because it is actually telling me that I am using it incorrectly”*.

(Patient 8)

#### 3.2.3. Acceptability of the Personalised INCA^TM^ Feedback

Perceived usefulness of the INCA^TM^ feedback

All interviewees considered the importance of INCA^TM^ technology. Hence, they had positive attitudes towards the information presented in the INCA^TM^ feedback and reported that the feedback was useful in terms of uncovering any gaps in their daily usage, whether related to adherence or IT, which would enable them to correct these gaps to ensure better usage in the future.

*“…it (referring to the feedback) gives me an insight of what I have been doing wrong, I should be able to correct it…”*.

(Patient 4)

In one case, the use of the feedback within the consultation identified the temporal patterns of inhaler use and uncovered that the patient was using it only for symptom relief rather than prevention.

*“…I was missing doses and not taking twice a day like regularly, up till now I was just using it when I needed it, you know, which was obviously wrong ……It (referring to the feedback) certainly told me. I mean this one the usage technique (referring to the graph) it seems to come up odd and messy for me, you know, it is all messy, I can see what you mean, it is three times there, twice, twice and then odd times…”*.

(Patient 9)

In another case, the patient indicated the utilisation of the alarm on their mobile phone as a reminder mechanism in order to promote their adherence level, upon seeing the results of the feedback.

*“… I was doing wrong and now I know exactly when to take it. I am going to put an alarm on my phone and then I can take it in the correct times. Hopefully it will be much better for my condition”*.

(Patient 17)

The feedback also raised the awareness of some patients about their own performance, as illustrated in the below quotation.

*“To prevent future symptoms, it made me more aware that I have to use it correctly now morning and evening”*.

(Patient 11)

The benefit of the INCA^TM^ feedback being visible objective evidence about adherence and IT over the current standard method, which is the IT checklist, was clearly demonstrated in one case where the patient was over relying/over using the reliever inhaler and not noticing any benefit, despite having their IT checked by HCPs at the pharmacy, respiratory unit, or pulmonary rehabilitation program. The patient highlighted that none of the HCPs who examined them were able to identify any problem in the technique on the spot, which subsequently led to a discussion about stepping up the treatment.

*“Yea. so I knew something wasn’t quite right especially coming with your question about the pharmacist because he mentioned this before, this doesn’t look right, overusing salbutamol but we didn’t really know what it was until you have a chart like this and put it in front of me and now it makes a lot of sense…. without this, literally the graph we are looking at it (the usage technique graph) now I don’t think we ever would have linked up, because what’s really interesting, now I am going in my mind thinking ahead so next month I have my annual check in a respiratory unit so there plus I did the pulmonary rehabilitation program ….but in both of those contexts they ask us to bring the inhalers and show how we use them, technique, so I am doing it in front of a professional so none of us have picked up on is what this picked up on, actually I am not quite clicking it till the end before I inhale, so none of the professionals or I picked up that this was happening so we would have gone for years thinking it was appropriately taken…”*.

(Patient 16)

This participant went a step further by showing concern in terms of healthcare costs because, in their particular context/case, using more reliever medication and stepping up the preventer treatment meant more costs to the NHS.

*“I love the NHS so I am conscious of cost…..if I am not using this properly (referring to the preventer) and I am offsetting the use of medication with another medication (referring here to the reliever) because this (pointing to the preventer) isn’t working so I am not only having the issue of not using the inhaler properly, then I am overusing another medication (the reliever) unnecessarily, so now we have two issues, one not being used properly and one being used improperly…”*.

(Patient 16)

The same participant then provided an additional comment about the benefit of the feedback as evidence about patients’ performance, especially when there is a poor relationship between the patient and the HCP due to the objective nature of the feedback.

*“…what’s the relationship like between the patient and the person delivering the message…… that’s why the graph works as well because if the relationship wasn’t good the graph is outside of that…”*.

(Patient 16)

Perceived ease of understanding of the INCA^TM^ feedback

The graphical representation of information about IT and adherence was preferred over the written information by all interviewees in the INCA^TM^ feedback. The participants reported that the feedback was easy to understand due to graphs which are easier to read and understand.

*“It (referring to the graphs) is just easier to read”*.

(Patient 18)

Other interviewees highlighted that the graphs provided a kind of an immediate visual impact and a detailed presentation about their performance at the same time.

*“…it makes it very clear that I am using it incorrectly, I think it is very very useful so it, you know, allows me immediately to see a visual, visual sort of information, allow you to sort of understand immediately where you need to get better, so I am happy with it”*.

(Patient 6)

Furthermore, the use of colours within the graphs facilitated and guided the comprehension of the information and made it easier to understand.

*“Yes very easy… because green and orange, the colours are telling me”*.

(Patient 2)

#### 3.2.4. Positive Perceptions about the Tailored Consultations

All patients appreciated the tailored consultation provided by the CP. All interviewees perceived the consultation to be beneficial and informative and highlighted some new learning out of it, either in terms of rectifying their IT or promoting their adherence level.

*“I learned that I wasn’t inhaling hard enough, I was not getting, I was putting my mouth too far over the rim instead of just of over the mouthpiece”*.

(Patient 18)

Analysis of the interviews also revealed that having a good relationship between the patients and the CPs also contributed to the positive perceptions about the consultations provided.

*“I thought I was doing it right but obviously I wasn’t but I mean this pharmacist here he has been so good to me, he always looked after me well and he has pointed out what to do so I have no concern, it is just my own fault I was not doing it right”*.

(Patient 7)

## 4. Discussion

The current results demonstrated significant improvements in patients’ actual adherence rate and TER, as identified by the INCA^TM^ device, while using the personalised feedback generated by the device during the discussions between the CPs and the patients. Provision of feedback is an important aspect in patient education and is considered a critical component of any educational intervention [22]. Currently, the nature of the feedback in evidence-based IT educational interventions is qualitative [22], mainly through evaluating IT against device-specific checklists. Nevertheless, emerging evidence in the literature highlights that the nature of the feedback has an impact on the effectiveness of IT education [22]. The study of Toumas-Shehata et al. [22] was the first to show that the use of qualitative and quantitative feedback for IT education was associated with higher scale of improvement in IT compared to qualitative feedback alone. In addition, the benefit of the INCA^TM^ feedback for educating patients was already established by the Irish researchers who developed it via two RCTs [20,21]. The two RCTs reported a significant difference in actual adherence rates between the control group (CG) and the group receiving education with feedback from the INCA^TM^ device [20,21]. Thus, a quite important and distinguishing aspect in the present study is the provision of consultations with personalised feedback about adherence and IT. In fact, the current strategies to improve adherence in respiratory conditions have received too little attention in Europe, despite the substantial clinical and economic burden of non-adherence [40]. Consequently, and considering this context, a report from the first European Congress on adherence to therapy was published in 2017 and highlighted the urgency to put non-adherence to inhaled respiratory medication higher on Europe’s policy agenda, with one way of achieving this being through the provision of tailored and personalised adherence-enhancing interventions [41]. Digital technologies targeting IT and adherence monitoring, such as the EMD mentioned in the current study, provide the opportunity to advance the level of personalised care offered to patients with chronic respiratory conditions [8,42].

Furthermore, the current study reinforces the findings of other studies [3,43,44,45,46,47,48], which showed that CPs are well suited to providing counselling and education about adherence and IT. The current study also comes in line with the existing evidence in the literature that has emerged in recent years regarding the benefits of EMDs in inhaler adherence monitoring and promoting adherence to inhaler therapy [14,15,16,17,18,19,20,21,22,23] and illustrates the value of embedding objective feedback measures within services aimed at medicine optimisation. Despite the paucity of robust evidence behind the effectiveness or cost-effectiveness of the MUR service in England [49,50], which led to it being phased out in the latest community pharmacy contractual framework 2019/20 to 2023/24, the case is not the same in Italy. In fact, the evidence generated from Italy via a RCT showed the Italian MUR (I-MUR) service for asthma patients to be effective at improving asthma control and adherence [49]. The Italian study also reported that the probability of the I-MUR intervention being more cost-effective was 100% compared to usual care [49]. Therefore, it can be concluded that the observed improvements in adherence and IT in the current study are due to a combined effect of the MUR consultation plus the use of the INCA^TM^ objective personalised feedback. Thus, the current research provides preliminary evidence, as limited by its sample size, for the value of focused community pharmacy medication reviews for respiratory patients and highlights the importance for pharmacists to consider tailoring these medication reviews to the needs of respiratory patients using objective evidence about adherence and IT, such as the one generated by the INCA^TM^ technology.

The interview data highlighted patients’ positive attitudes towards the INCA^TM^ technology and the consultations provided by the CPs. Interestingly, this was further supported by the feedback questionnaire results. All the interviewed patients were receptive to the idea of monitoring adherence and IT. This resonates with the findings of other studies [1,6], which showed patients to have positive attitudes towards adherence monitoring and reminders using the SmartTrack device, which is an EMD for monitoring inhaler adherence only [1,6].

In the current study, the interview data revealed patients’ acceptance of and willingness to use the INCA^TM^ device in the future. The perceived acceptability by patients in the current study can be explained by the technology acceptance model (TAM), alongside the integrative review of Gucin and Berk [51] focusing on the factors affecting technology acceptance by HCPs and patients. In TAM, perceived usefulness and perceived ease of use are the two main factors affecting the technology usage of individuals [51]. Similarly, the integrative review of Gucin and Berk [51] indicated that both perceived benefits and perceived ease of use are the most influential variables on technology acceptance for patients in healthcare. Regarding perceived usefulness and benefits of the INCA^TM^ technology, the interview data indicated that patients wanted to use the INCA^TM^ device again and were in favour of recommending it to friends or relatives. During the interviews, some of the patients elaborated how they perceived their own performance to be non-erroneous, especially in terms of usage technique, which was not the case as identified by the INCA^TM^ device; hence, they recommended the INCA^TM^ device to other patients who might have similar perceptions about their performance to raise other patients’ awareness about inhaler usage. The interviewees also highlighted their willingness to use the INCA^TM^ device again to get further feedback about their performance after the consultation to ensure that they are using their inhalers correctly. Additionally, the questionnaire results highlighted that the majority of patients considered the INCA^TM^ feedback to be useful during the discussions. Interestingly, the interview data largely reinforced these findings and elaborated more on the reasons behind this. The interviewees found the INCA^TM^ feedback to be useful due to several reasons, including identifying errors and gaps in inhaler usage to rectify, being objective paper-based evidence about performance, raising their awareness about adherence and technique, promoting change in adherence attitudes through habit formation as in the case of employing alarms, and potentially providing cost savings for the NHS. With respect to perceived ease of use, the questionnaire results indicated that the adapted accuhaler was perceived as either very easy or easy to use by all patients, except one. In addition, the handling of the adapted accuhaler was perceived as either manageable or very manageable by all patients. It is worth mentioning that, after mounting the INCA^TM^ device on the accuhaler, there is no further interaction required from patients except using the mounted accuhaler as normal, which might also have positively affected patients’ attitudes about the ease of use. The questionnaire results also indicated that all patients found the INCA ^TM^ feedback to be either very easy or easy to understand, and this was backed up with the interview data, which showed that the INCA^TM^ feedback was easy to understand due to the inclusion of coloured graphs providing a direct visual representation of adherence and IT. Interestingly, this mirrors the findings of Foster et al.’s [1] study, which showed that most participants found the adherence graphs generated by the SmartTrack device to be easy to understand. In fact, graphs can be considered an appealing alternative to numerical information because they are visually interesting and stimulate automatic visual perception skills; moreover, they are often used in print and electronic formats to educate patients [52].

All the interviewed patients also perceived the consultations delivered by the CP as beneficial. This is further corroborated by the quantitative results of the service evaluation, which showed significant improvements in the participants’ actual adherence rate and TER as identified by the INCA^TM^ device after the discussion with the CP.

### Strengths and Limitations

To the best of our knowledge, this is the first study in England that involved patients having consultations with their CP whilst using objective and personalized feedback about IT and adherence. Furthermore, this is the first study to explore patients’ perceptions and acceptance of the INCA^TM^ device, an EMD that monitors both adherence and IT at the same time.

Although the current study had positive results, limitations in data interpretation and generalisability can yet be attributed to the following factors: short duration of the study, selection bias, patients being from an urban community in South and West London and using only one type of DPI, and the absence of CG and the possibility of Hawthorne effect due to patients’ awareness about the INCA^TM^ device. Although recruited patients were regular customers to the pharmacies, the likelihood of receiving advice or training about adherence and IT from another pharmacy cannot be excluded during the course of the study. In addition, the quality of discussions delivered by CPs may have varied according to the CPs’ skills and experience. The small sample size is another factor which affects the validity and the reliability of the results and limits its generalisability. However, the current study did not aim to investigate any correlational relationship or detect differences between group populations but aimed at shedding the light on the value of embedding objective feedback about inhaler adherence within patients’ consultations using a new technology; the sample size was also restricted due to the researchers’ available time, logistical reasons, and limited resources.

## 5. Conclusions

The present study, albeit with a small sample size, adds to the existing evidence in the literature regarding the positive impact of CPs in supporting respiratory patients with IT and adherence and the potential of assistive technologies in optimising services targeted at medicine optimisation among patients with long term conditions. The current study demonstrated significant improvements in patients’ adherence and IT through the provision of tailored consultations using objective feedback. Patients perceived positive benefits from the technology mainly in terms of providing personalised visual feedback about performance, thus identifying potential gaps and patterns of inhaler usage. The perceived benefits led to positive attitudes among patients towardsd the use of the INCA^TM^ device for performance monitoring, and towards recommending its use to other patients as well.

## Figures and Tables

**Table 1:** Demographic characteristics for patients with available acoustic data (*n* = 18), before and after the service evaluation.

Characteristics	Frequency (%)	Median (IQR)
Age (y)		64.5 (20.3)
Gender, *n* (%)		
Female	10 (56)	
Male	8 (44)	
Respiratory condition		
Asthma	6 (33%)	
COPD	12 (67%)	
Smoking history		
Non-smoker	5 (28)	
Current smoker	5 (28)	
Ex-smoker	8 (44)	
Education Level		
Primary	1 (6)	
Secondary	7 (39)	
College	6 (33)	
Undergraduate	2 (11)	
Postgraduate	2 (11)	
Marital status		
Single	5 (28)	
Married	8 (44)	
Divorced	1 (6)	
Widow/Widower	4 (22)	
Residential area		
Kingston Upon Thames	2 (11)	
Richmond Upon Thames	6 (33)	
Wandsworth	1 (6)	
Merton	6 (33)	
Sutton	3 (17)	
Flu vaccination		
Yes	17 (78)	
No	4 (22)	
Pneumococcal vaccination		
Yes	7 (39)	
No	11 (61)	
Presence of comorbidities		
Yes	15 (83)	
No	3 (17)	
No. of comorbid conditions		2 (1)
No. of medications per month		10 (7)
No. of GP visits per year due to COPD or asthma		2.5 (3)
Exacerbations due to COPD or asthma during the last year		
Yes	10 (56)	
No	8 (44)	
No. of exacerbations due to COPD or asthma during the last year		2.5 (3)
No. of exacerbations due to COPD during the last year		0.5 (2)
No. of exacerbations due to asthma during the last year		2 (3)
A & E visits due to COPD or asthma during the last year		
Yes	1 (6)	
No	17 (94)	
No. of A & E visits due to COPD or asthma during the last year		3 (0)
Hospital admissions due to COPD or asthma during the last year	3 (17)	
No	15 (83)	
No. of hospital admissions due to COPD or asthma during the last year		1 (0)

**Table 2:** Breakdown of audio files/doses for the observed patients before and after the consultation with the CP.

	Number of Audio Files (before)	Number of Audio Files (after)	*p* Value
Expected doses	1080	1080	/
Attempted doses for >30 days, electronic doses with evidence of priming	999	1077	0.628
Attempted doses for 30 days, electronic doses with evidence of priming	830	907	0.421
Actual doses for 30 days, accounting for missed doses, and incorrect technique	396	726	0.002 **

** Indicate statistical significance of the result at *p* < 0.01.

**Table 3:** Frequency and percentage of patients showing ≥ or <80% attempted and actual adherence, before and after the consultation with the CP.

INCA^TM^ Adherence Measures	Patients Having ≥ 80% Adherence before, *n* (%)	Patients Having ≥ 80% Adherence after, *n* (%)
INCA^TM^ attempted adherence	4 (22)	10 (56)
INCA^TM^ actual adherence	0 (0)	4 (22)

**Table 4:** The type of different inhalation errors observed before and after the consultation with the CP.

Audio Error	Number (%) of Audio Files before (*n* = 434)	Number (%) of Audio Files after (*n* = 181)	*p* Value
**Errors in drug priming/drug blistering**			
No drug priming, inhalation detected	75 (17.3%)	45 (24.9%)	0.268
Multiple drug priming	6 (1.4%)	4 (2.2%)	0.480
Multiple drug priming and multiple inhalation	3 (0.7%)	2 (1.1%)	0.705
Dose dumping	0	0	
**Total**	**84**	**51**	
**Errors in inhalation**			
Exhales into the inhaler after drug priming and before inhalation	10 (2.3%)	3 (1.6%)	0.336
Drug priming present, no subsequent inhalation detected	124 (28.6%)	43 (23.8%)	0.277
Multiple inhalations	216 (49.7%)	84 (46.4%)	0.009 **
**Total**	**350**	**130**	

** Indicate statistical significance of the result at *p* < 0.01.

**Table 5:** The TER among the observed patients before and after the consultation with the CP.

TER	Number of Patients Before (Percentage)	Number of Patients after (Percentage)
TER < 20%	4 (22)	12 (67)
TER ≥ 20%	14 (78)	6 (33)
Total	18 (100)	18 (100)

Abbreviations: TER, Technique Error Rate.

**Table 6:** Emergent themes and subthemes from patients’ interviews.

Themes	Subthemes
Theme 1: Acceptability of the INCA^TM^ technology	Subtheme 1: Patients’ positive perceptions about the INCA^TM^ technology. Subtheme 2: Recommendation of the INCA^TM^ device to other patients.
Theme 2: Patients’ misperceptions about their inhaler usage	None
Theme 3: Acceptability of the personalised INCA^TM^ feedback	Subtheme 1: Perceived usefulness of the INCA^TM^ feedback. Subtheme 2: Perceived ease of understanding of the INCA^TM^ feedback.
Theme 4: Positive perceptions about the tailored consultations	None

## Data Availability

The data presented in this study are available on request from the corresponding author. The data are not publicly available due to legal and privacy restrictions.

## Supplementary Materials

The following supporting information can be downloaded at: https://www.mdpi.com/article/10.3390/pharmacy11030094/s1, File S1: patient satisfaction questionnaire; File S2: Interview schedule/topic guide for patients after the consultation with the CP.
