# Supplementary material for: Objective Assessment of Adherence and Inhaler Technique among Asthma and COPD Patients in London: A Study in Community Pharmacies Using an Electronic Monitoring Device
Source: Pharmacy (Basel). 2023 Jun 4;11(3):94. doi: 10.3390/pharmacy11030094 (PMC10304614; doi:10.3390/pharmacy11030094)
Supplement: Supplementary file 1 [file pharmacy-11-00094-s001.zip › Supplementary file 2-The interview topic guide for patients.pdf]

Supplementary file 2: Interview schedule/topic guide for patients after the consultation with the CP

Section a: about the consultation with the CP

1- How do you feel about the session you had with your pharmacist? Why?

2- Could you please tell me about the content of the session? (i.e.: What was discussed during the session)

> Prompt: Information about COPD/asthma as a disease, side-effects of your medications, how to use your Seretide inhaler, how to use your other inhalers, how to use your other medications for other conditions, all of the above, none of the above, others)

3- How did the pharmacist explain to you about the way of using your inhaler?

> Prompt: written instructions only, verbal instructions only, written and verbal instructions, or verbal instructions and demo.

4- What did you learn as a result of the discussion you had with the pharmacist?

> Prompt: if no learning was achieved, then ask why the patient could not understand everything discussed during the session.

Section b: about the INCA™ device and feedback

5- What do you think about the information presented in the feedback that was generated by the INCA™ device?

Prompt:

- Was it useful or not? Why?
- Was it easy to understand or not? Why?
- What was the most useful piece of information in the feedback discussed? Why?

6- Would you consider using this device again and get feedback about how you use your inhaler? Why?

1- How do you feel about recommending this device to friends and relatives who also use inhalers? Why?

Section c: suggestions for improving the consultation with the CP

8- What was the most benefit you gained out of this discussion?

9- How would you improve this discussion?

> Prompts: more time, easier language, more information, consultation style of the pharmacist.

Further additions/ comments:

Is there anything else you would like to share with me regarding the discussion you had with your pharmacist?
