# Supplementary material for: Objective Assessment of Adherence and Inhaler Technique among Asthma and COPD Patients in London: A Study in Community Pharmacies Using an Electronic Monitoring Device
Source: Pharmacy (Basel). 2023 Jun 4;11(3):94. doi: 10.3390/pharmacy11030094 (PMC10304614; doi:10.3390/pharmacy11030094)
Supplement: Supplementary file 1 [file pharmacy-11-00094-s001.zip › Supplementary file1-The study questionnaire.pdf]

Supplementary file 1: Patient satisfaction questionnaire

Code:

Patient feedback questionnaire:

Dear participant,

Thank you very much for participating in our study, we would like to get your feedback about your experience in the study. Please note that responses will be kept strictly confidential, and will not be shared even with your healthcare team.

Section 1:

Could you please tell me about your experience with using INCA^{TM} technology regarding the following:

1- The use of the adapted inhaler (the inhaler with the INCA^{TM} device)

Very easy	Easy	Neither easy nor difficult	Difficult	Very difficult
1	2	3	4	5

2- The handling of the adapted inhaler (the inhaler with the INCA^{TM} device)

Very manageable	Manageable	Undecided	cumbersome	Very cumbersome
1	2	3	4	5

Section 2:

Please rate the ease of the following:

3- The **information** provided by the pharmacist about **my inhaler use**

Very easy	Easy	Neither easy nor difficult	Difficult	Very difficult
1	2	3	4	5

4- The **feedback** discussed with the pharmacist about **my inhaler use**

Very easy	Easy	Neither easy nor difficult	Difficult	Very difficult
1	2	3	4	5

5- The **consultation style** of the pharmacist

Very easy	Easy	Neither easy nor difficult	Difficult	Very difficult
1	2	3	4	5

6- The **language** used by the pharmacist during the discussion/consultation

Very easy	Easy	Neither easy nor difficult	Difficult	Very difficult
1	2	3	4	5

Section 3:

Please rate the usefulness of the following:

7- The **information** provided by the pharmacist about **my inhaler use** was useful

Strongly agree	Agree	Neither agree nor disagree	Disagree	Strongly disagree
1	2	3	4	5

8- The **feedback** discussed with the pharmacist about **my inhaler use** was useful

Strongly agree	Agree	Neither agree nor disagree	Disagree	Strongly disagree
1	2	3	4	5

Section 4:

Please rate your satisfaction with the following:

9- Time spent with the pharmacist during the discussion

Very satisfied	Satisfied	Neither satisfied nor dissatisfied	Dissatisfied	Very dissatisfied
1	2	3	4	5

10- Information provided during the consultation

Very satisfied	Satisfied	Neither satisfied nor dissatisfied	Dissatisfied	Very dissatisfied
1	2	3	4	5

11- Feedback provided during the consultation

Very satisfied	Satisfied	Neither satisfied nor dissatisfied	Dissatisfied	Very dissatisfied
1	2	3	4	5

12- Overall satisfaction with the discussion received by the pharmacist

Very satisfied	Satisfied	Neither satisfied nor dissatisfied	Dissatisfied	Very dissatisfied
1	2	3	4	5

13- Please provide any further comments you want to share with us

----

Thank you for taking time to complete this questionnaire.
